# Digital self-presentation and adolescent mental health: Cross-sectional and longitudinal insights from the “LifeOnSoMe”-study

**DOI:** 10.1186/s12889-024-20052-4

**Published:** 2024-09-27

**Authors:** Gunnhild Johnsen Hjetland, Turi Reiten Finserås, Børge Sivertsen, Ian Colman, Randi Træland Hella, Amanda Iselin Olesen Andersen, Jens Christoffer Skogen

**Affiliations:** 1 Department of Health Promotion, Norwegian Institute of Public Health, Bergen, Norway; 2 Centre for Evaluation of Public Health Measures, Norwegian Institute of Public Health, Oslo, Norway; 3 Department of Research and Innovation, Helse Fonna HF, Haugesund, Norway; 4 School of Epidemiology and Public Health, University of Ottawa, Ottawa, Canada; 5 Centre for Fertility and Health, Norwegian Institute of Public Health, Oslo, Norway; 6 Regional Centre for Child and Youth Mental Health and Child Welfare, NORCE Research, Bergen, Norway; 7 Alcohol and Drug Research Western Norway, Stavanger University Hospital, Stavanger, Norway

**Keywords:** Social media, Adolescent, Mental health, Self-presentation, Upward social comparison

## Abstract

**Background:**

The intensive use of social media among adolescents has caused concern about its impact on their mental health, but studies show that social media use is linked to both better and worse mental health. These seemingly contradictory findings may result from the diverse motivations, interactions, and experiences related to social media use, and studies investigating specific facets of social media use in relation to mental health and well-being, beyond general usage metrics, have been called for. Aspects of self-presentation on social media, such as feedback-seeking and upwards social comparison have been linked to worse mental health, however, there is a need for more studies exploring the relationship between self-presentation on social media and adolescent mental health over time.

**Aim:**

The aim of this study was to explore the cross-sectional and longitudinal relationship between aspects of self-presentation and depression, anxiety, and well-being among adolescents.

**Methods:**

This study utilised both cross-sectional and longitudinal datasets from the LifeOnSoMe-study, comprising 3,424 and 439 participants, respectively (OSF preregistration 10.17605/OSF.IO/BVPS8). Latent Class Analysis (LCA) was used to identify similar response patterns within the Self-Presentation and Upwards Social Comparison Inclination Scale (SPAUSCIS). Regression models and first differencing methods were applied to evaluate the cross-sectional and longitudinal associations between focus on self-presentation and mental health and well-being among adolescents.

**Results:**

A strong emphasis on self-presentation was linked to increased levels of depression and anxiety in both males and females, and reduced well-being in females when compared to those with lower or intermediate self-presentation focus. The effect sizes ranged from small to medium. Furthermore, an escalation in self-presentation focus over time was associated with a slight increase in symptoms of anxiety and depression; however, the association with well-being did not reach statistical significance.

**Conclusion:**

The results of the present study suggest that a heightened focus on self-presentation, which includes behaviours such as seeking feedback, employing strategic self-presentation tactics, and engaging in upward social comparisons, is associated with an elevated risk of reduced mental health.

**Supplementary Information:**

The online version contains supplementary material available at 10.1186/s12889-024-20052-4.

## Introduction

The intensive use of social media among adolescents has caused concerns about its impact on their mental health and well-being, and a host of scientific papers have addressed this issue [1]. A recent umbrella review showed that the amount of time spent on social media use is weakly associated with both higher levels of mental health problems and with higher well-being among adolescents [2]. These seemingly contradictory findings may be attributed to heterogeneity of social media use and to person-specific effects [3], meaning that social media use can entail widely different motivations, interactions, experiences, and behaviours, and that any effects of social media use are likely to vary depending on how, why, and by whom they are used. Therefore, investigating how particular facets of social media use influence mental health and well-being, beyond general metrics of frequency and duration of use, has been called for [2]. In addition, research should focus on key attributes spanning a range of different social media platforms, in line with an affordance approach [4], to stay relevant in the ever evolving social media landscape. In the context of social media, affordances refer to “the perception of action possibilities users have when engaging with social media and its features” ([5], pp. 408–409).

One aspect of social media use that has been studied in relation to mental health and well-being is self-presentation [6, 7]. Social approval is seen as one of the main goals of self-presentation on social media [8], and some adolescents place great emphasis on their online personas [9–11]. In line with Goffman’s theory of self-presentation and social interaction [12], all social encounters entail some form of performance to manage how one is perceived by others (i.e., self-presentation). To present the best possible version of themselves, people downplay certain characteristics and enhance others; a process called impression management [12]. Compared to traditional face-to-face interactions, social media affordances facilitate impression management and idealized self-presentation by allowing users to manipulate their text and image based communication [13]. Furthermore, the number of likes and number or content of comments can easily be compared to others’ to quantify one’s social success [14]. Some people make great efforts to receive the desired feedback, referred to as feedback-seeking or digital status seeking [15]. Arguably, as self-presentation on social media is often idealized and is mainly positive, upward social comparison, i.e., comparing oneself to someone who is viewed as better than oneself [16], may be particularly likely [14, 17]. Social media use also increases the number of available comparison targets to include not only peers in one’s immediate surroundings, but also a wider network of acquaintances, ‘influencers’, and celebrities, thereby expanding the opportunities for engaging in upward social comparison.

Studies on adolescents have shown that different aspects of self-presentation, such as feedback-seeking, strategic self-presentation such as editing photos, and upward social comparison, are associated with worse mental health in terms of more symptoms of anxiety and depression, and reduced body satisfaction and well-being [11, 18–21]. These findings can be linked to the broader concept of ‘approval anxiety’, i.e., the degree of psychological arousal about others’ reactions to one’s messages and posts on social media, which has been proposed as one component of digital stress [22]. Digital stress, in turn, has been shown to increase the risk of negative mental health outcomes as a result of social media use. Self-presentation on social media may therefore be one aspect of social media use that can have negative consequences for adolescent mental health. Most previous studies are, however, based on cross-sectional data, and more longitudinal studies are needed to establish the relevance of aspects of self-presentation on social media to adolescent mental health. The few longitudinal studies that exist have shown that posting a lot of content on social media, being preoccupied with one’s physical attractiveness in social media photos, feedback-seeking, and upward social comparison are linked to symptoms of anxiety and depression, and reduced well-being [18, 23, 24].

Adolescence is a period when peer approval becomes increasingly relevant, and seeking approval alongside a heightened sensitivity to social rewards may be a an important motivator for using social media during this developmental phase [5]. Adolescents seem to vary a great deal in their preoccupation with self-presentation on social media. In a previous study, we investigated how adolescents differed in their preoccupation with likes, comments, and followers, in deleting posts with too few likes and manipulating images to look better, and in upward social comparison, collectively referred to as “focus on self-presentation” [25]. The results showed that females and adolescents with low emotional stability and high scores on extraversion, were more likely to be highly focused on self-presentation. Similarly, adolescent girls have been found to report higher levels of feedback-seeking and social comparison [11, 18], post more ‘selfies’, be more focused on their physical appearance, and be more concerned about peer feedback, compared to adolescent boys [26]. While some research has found that the associations between aspects of self-presentation on social media and mental health problems are similar for boys and girls [24], some findings indicate that the association is stronger for girls [11, 18].

The aim of the present study was to further explore the relationship between focus on self-presentation and depression, anxiety, and well-being. Firstly, a latent class analysis was used to map out response patterns on a seven-item scale assessing focus on self-presentation among adolescents. Secondly, the association between these response patterns and mental health was assessed separately for males and females using a cross-sectional dataset. Lastly, the longitudinal association between focus on self-presentation and mental health was assessed over two time-points.

## Methods

The present study (OSF preregistration 10.17605/OSF.IO/BVPS8) was based on data from an online survey conducted in two rounds in 2020 and 2021, in Bergen, Norway, called the LifeOnSoMe-study. Bergen is the second-largest city in Norway and has a population of about 300,000. All senior high school pupils of 16 years or older were invited to participate in the survey via their teachers and information screens on their school. The pupils received a link to a website and logged in using their electronic ID. Before starting the survey, they received information about the study and provided their informed consent. In addition, those that had participated in the 2020 data collection received an email with a link to the survey. The participation rate was 53% in 2020 and 35% in 2021. The broader aim of the LifeOnSoMe-study was to explore the relationship between adolescents’ motivations, experiences, and behaviours related to social media use and sociodemographic variables, lifestyle and social factors, and mental health.

The present study was based on two separate datasets. The cross-sectional dataset comprised responses from the two rounds of the survey. For those who completed the survey both in 2020 and 2021 (*n* = 461), we only used their 2020 responses. The total number of participants in the cross-sectional dataset was 3,771. Of these, participants missing information about gender (*n* = 5) or age (*n* = 158) were excluded. Furthermore, only 40 participants ticked the option “non-binary” for gender. This number is too low to perform meaningful analyses, and these participants were excluded from the study. Those with 100% missing values on the independent variable (*n* = 144) were excluded from the analyses, resulting in a total sample size of *n* = 3,424. The longitudinal dataset was based on the responses of those who completed the survey both in 2020 and 2021 (*N* = 461, 59% females). Of these, 22 participants missing 100% of the items of the independent variable were excluded (*n* = 4 at T1 and *n* = 18 at T2), resulting in a total sample of *n* = 439.

### Variables

#### Focus on self-presentation

To assess focus on self-presentation on social media, we used the Self-Presentation and Upward Social Comparison Inclination Scale (SPAUSCIS), which was developed based on qualitative focus group interviews with adolescents. The development of the scale is described in detail elsewhere [11, 25]. In a previous study, we showed that the SPAUSCIS had one latent factor and high internal consistency in a sample of adolescents [25]. The scale consists of 7 statements regarding focus on self-presentation on social media, covering feedback-seeking, strategic self-presentation, and upward social comparison (see supplementary Table S1). The participants were asked how much each statement pertained to them, and the response options were “not at all”, “very little”, “sometimes/partly true”, “a lot”, and “very much”, coded 1–5. The total score was computed by averaging the sum score on the total number of items, resulting in a total score ranging from 1–5. Cronbach’s alpha was 0.87 in the cross-sectional sample and 0.86 in the longitudinal sample (at T1).

#### Social media use

The participants’ frequency of social media use was measured by the following question: “How often do you use social media?” The response alternatives were “almost never”, “several times a month, but less than once a week”, “1–2 times per week”, “3–4 times per week”, “5–6 times per week”, “every day”, “several times each day”, and “almost constantly”. In the present study, we created a tripartite variable which differentiated between “daily or less”, “many times each day”, and “almost constantly”. The participants’ duration of social media use was assessed by the following question: “On the days that you use social media, approximately how much time do you spend on social media?” The seven response options ranged from “less than 30 min” to “more than 5 h”. The response options were categorized into “less than 2 h”, “2–4 h”, “4–5 h”, and “more than 5 h”.

#### Symptoms of anxiety

Symptoms of anxiety were measured using the General Anxiety Disorder 7 (GAD-7; [27]). The GAD-7 consists of 7 questions related to symptoms of general anxiety. The response options ranges from 0 (not at all) to 3 (almost every day). The measure was used as a continuous variable with the total score ranging from 0 to 21. Cronbach’s alpha was 0.90 in the cross-sectional sample and 0.89 in the longitudinal sample (at T1).

#### Symptoms of depression

Symptoms of depression were measured using the Short Mood and Feelings Questionnaire (SMFQ; [28]). The SMFQ consists of 13 statements related to symptoms of depression. The response options are 0 (not true), 1 (sometimes true), and 2 (true). The scores on each item are summed to a total score ranging from 0 to 26. The measure was used as a continuous variable. Cronbach’s alpha was 0.91 in the cross-sectional sample and 0.88 in the longitudinal sample (at T1).

#### Well-being

The Warwick-Edinburgh Mental Well-Being Scale (WEMWBS) was used to assess the participants’ level of mental well-being [29]. The WEMWBS focuses solely on positive aspects of mental health, covering positive affect, satisfying personal relationships, and positive functioning. The scale has 14 positively scored items and responses are given on a 5-point Likert scale ranging from “none of the time” (1) to “all of the time” (5). The minimum score is 14 and the maximum score is 70, with a higher score indicating better mental well-being. The responses are based on the previous two weeks. The Norwegian version of the WEMWBS was used in the present study, which has shown good validity and reliability for Norwegian adolescents [30]. Cronbach’s alpha was 0.93 in the cross-sectional sample and 0.92 in the longitudinal sample (at T1).

#### Background variables

Participants provided their age, gender, and which year in senior high school (first, second, or third) and which program they attended college (preparatory or vocational education). Subjective socioeconomic status (SES) was assessed by the question “How well off do you consider you own family to be compared to others?” The response options ranged from 0 (“very poor”) to 10 (“very well off”). In the current study, SES was recoded into a tripartite variable of low SES (scores 0–4; 6.4%), medium SES (5–7, 52%), and high SES (8–10, 42%). Personality was measured using the Ten-Item Personality Inventory [31], consisting of ten items measuring two opposing traits of each personality dimension (Extraversion, agreeableness, conscientiousness, emotional stability, and openness to new experiences). The items are preceded by “I see myself as”, followed by trait adjectives. The response categories range from 1 (strongly disagree) to 7 (strongly agree). The total score on each trait is calculated by taking the average of the two items after recoding the reverse-scored item, resulting in a total score ranging from 2 to 14.

### Statistical analyses

All analyses were performed using R version 4.1.3 [32] and RStudio version 2023.06.1 + 524 [33]. To assess the structural validity of the SPAUSCIS, a confirmatory factor analysis was performed using the cross-sectional dataset. Internal validity was assessed with Cronbach’s alpha, using the ‘psych’ package [34] and the confirmatory factor analysis was performed using the ‘lavaan’ package [35] and DWLS estimator suitable for ordinal variables [36]. Groups with similar response patterns on the items of the SPAUSCIS were identified using latent class analysis (LCA), using the ‘poLCA’ package [37]. The most appropriate number of latent classes was chosen based on several statistical criteria: Aikake information criterion (AIC), Bayesian information criterion (BIC), relative entropy, and the Lo-Mendell-Rubin ad hoc adjusted likelihood ratio test (LMR-LR), as well as interpretability of the model.

#### Cross-sectional associations

Linear regression was used to assess the associations between latent class membership and depressive symptoms, symptoms of anxiety, and well-being. The associations were estimated for the full sample and separately for males and females, and expressed as coefficients with corresponding standard errors, in addition to Cohen’s ds. As SES, frequency and duration of social media use, and the personality traits of extraversion and emotional stability has been linked to both focus on self-presentation [25] and to mental health outcomes in previous studies [2, 38, 39], all regressions were adjusted for these variables in multiple linear models. For the full sample, adjustments were also made for gender. Adjusted Cohen’s *d* values were calculated following the procedure included in the ESIZEREG module for Stata [40]. Likelihood ratio tests were used to examine a potential gender moderation in the associations between class membership and the dependent variables, comparing models with the interaction gender × class membership and models with gender included as a covariate. In all analyses, a *p*-value of < 0.05 indicated statistically significant associations. A post-hoc analysis assessing the correlation between the total score of the SPAUSCIS as a continuous variable with symptoms of depression and anxiety, and well-being using Spearman rank correlation.

#### Longitudinal associations

The ‘plm’ package [41] was used to estimate first difference models to assess the longitudinal associations between focus on self-presentation and mental health and well-being. First difference models difference out fixed effects such as gender, socioeconomic status and other variables that are assumed to be fixed over time [42]. Thus, the model avoids bias due to unobserved time-invariant variables. To ease interpretation of the results, we report both the “raw” coefficients and coefficients based on z-scored dependent variables. When using standardized dependent variables, the coefficients are interpreted as standard deviations: For every one-unit increase in the independent variable, the dependent variable increases by a given number of standard deviations.

### Missing data

There were some missing data. After excluding those that were missing 100% of the SPAUSCIS items from the dataset, there were 0.8 to 3.9% missing on the items of the SPAUSCIS in the cross-sectional dataset, 0.2 to 1.1% missing in the longitudinal dataset at T1 and 1.1 to 4.3% at T2. The SPAUSCIS total score is calculated as the mean of the item scores and those missing one or more items received a mean based on the completed items.

The total scores of SMFQ, GAD-7 and WEMWBS were calculated by dividing the sum score of completed items on the number of completed items, multiplied by the total number of items of the relevant scale. Pairwise deletion was used throughout the analyses to retain as much information as possible.

## Results

Table 1 shows descriptive information for the cross-sectional data. The mean age of the sample was 17.28 years (SD 1.01), and 56% were girls. There were significant differences between girls and boys in all variables except age, school year and birth country. Females had higher scores on the duration and frequency of social media use and on focus on self-presentation, as well as on symptoms of depression and anxiety, and lower scores on well-being.

**Table 1:** Descriptives for the cross-sectional data

	Male (*N* = 1508)	Female (*N* = 1916)	Total (*N* = 3424)	*P* value
**Age**				0.738^a^
Mean (SD)	17.27 (0.98)	17.28 (1.00)	17.28 (0.99)	
**Year of high school**				0.035^b^
1	292 (19.5%)	402 (21.0%)	694 (20.4%)	
2	728 (48.5%)	842 (44.1%)	1570 (46.0%)	
3	480 (32.0%)	666 (34.9%)	1146 (33.6%)	
**Study program**				< 0.001^b^
College preparatory	1025 (68.4%)	1529 (79.8%)	2554 (74.8%)	
Vocational education	473 (31.6%)	386 (20.2%)	859 (25.2%)	
**Country of birth**				0.084^b^
Norway	1383 (91.9%)	1728 (90.2%)	3111 (90.9%)	
Other country	122 (8.1%)	188 (9.8%)	310 (9.1%)	
**Subjective socioeconomic status**				< 0.001^b^
Low (0–4)	65 (4.4%)	147 (7.7%)	212 (6.3%)	
Medium (5–7)	700 (47.1%)	1063 (56.0%)	1763 (52.1%)	
High (8–10)	721 (48.5%)	689 (36.3%)	1410 (41.7%)	
**Social media frequency**				< 0.001^b^
Daily or less	460 (30.5%)	364 (19.0%)	824 (24.1%)	
Many times each day	716 (47.5%)	984 (51.4%)	1700 (49.7%)	
Almost constantly	330 (21.9%)	568 (29.6%)	898 (26.2%)	
**Social media duration**				< 0.001^b^
< 2 h	565 (37.7%)	448 (23.5%)	1013 (29.7%)	
2–4 h	559 (37.3%)	736 (38.6%)	1295 (38.0%)	
4–5 h	200 (13.3%)	414 (21.7%)	614 (18.0%)	
> 5 h	176 (11.7%)	311 (16.3%)	487 (14.3%)	
**Anxiety, GAD-7**				
Mean (SD)	4.10 (4.39)	7.10 (4.98)	5.78 (4.96)	< 0.001^a^
**Depression, SMFQ**				
Mean (SD)	5.02 (5.03)	8.99 (6.36)	7.24 (6.14)	< 0.001^a^
**Well-being, WEMWBS**				
Mean (SD)	51.53 (9.70)	46.04 (9.62)	48.46 (10.03)	< 0.001^a^
**Focus on self-presentation, SPAUSCIS**				
Mean (SD)	1.54 (0.64)	2.21 (0.80)	1.91 (0.81)	< 0.001^a^

*GAD-7* General Anxiety Disorder 7, *SMFQ* Short Mood and Feelings Questionnaire, *SPAUSCIS* Self-presentation and Upward Social Comparison Inclination Scale, *WEMWBS* Warwick-Edinburgh Mental Well-Being Scale

^a^Linear model ANOVA

^b^Pearson’s Chi-squared test

The CFA of the items of the SPAUSCIS resulted in a Comparative Fit Index (CFI) of 0.999, a Tucker-Lewis Index (TLI) of 0.998, a root mean square error of approximation (RMSEA) of 0.051 (95%CI 0.043–0.060, *p* = .398), and a standardized root mean square residual (SRMR) of 0.021, all signalling god fit [43]. Items 2 and 3 and items 6 and 7 had highly correlated error terms, which were allowed for in the model.

The LCA yielded three classes corresponding to a low (class 1), intermediate (class 2), and high (class 3) focus on self-presentation, in line with the previous findings [25]. Predicted class membership was 44% in class 1, 33% in class 2, and 23% in class 3. Class 3 and 2 was dominated by females, while class 1 was dominated by males. Class 3 also had a lower proportion of adolescents with high SES, and a higher proportion of adolescents using social media ‘almost constantly’ compared to class 1 and 2. See supplementary figure S1 and table S2 for a more detailed description of the LCA results. See also supplementary table S3 for descriptives across class membership and S4 for an overview of SPAUSCIS scores across class membership.

Table 2 shows the results of the linear models. Being in class 3 was associated with higher symptoms of anxiety and depression compared to class 1 and 2 in both crude and fully adjusted cross-sectional analyses for the sample as a whole and for males and females when analysed separately (all p’s < .01). The effect sizes were small-to-medium in crude models (Cohen’s *d*s from 0.34–0.66 for anxiety and 0.43-0.74 for depression) and small in fully adjusted models (Cohen’s *d*s from 0.16–0.32 for anxiety and 0.25–0.33 for depression). For well-being, being in class 3 was associated with lower well-being compared to class 1 and 2 for males, females, and the sample as a whole in the crude models (all p’s < .05), with small effect sizes (Cohen’s *d* from -0.20- -0.46). In the fully adjusted models, the lower well-being associated with class 3 membership was no longer significant for males. Class 2 membership was not associated with any difference in symptoms of anxiety, depression or well-being compared to class 1 membership in adjusted models, but was associated with lower well-being for the sample as a whole in the crude model (Cohen’s *d* -0.13, *p* < .001). The likelihood ratio tests comparing models with and without the interaction term class membership × gender were not significant, meaning that the associations between class membership and anxiety, depression, and well-being were not significantly different for males and females (results provided in the Appendix, all p’s > .05).

**Table 2:** Linear models for GAD-7, SMFQ, and WEMWBS separate for males and females and for males and females combined

	Class 2 vs class 1, β (SE)	Cohen’s d	Class 3 vs class 1, β (SE)	Cohen’s d	Class 3 vs class 2, β (SE)	Cohen’s d
GAD-7 crude						
Males	0.37 (0.25)	0.09	2.62(0.40)***	0.59	2.25 (0.42)***	0.52
Females	0.20 (0.27)	0.04	1.87 (0.29)***	0.37	1.67 (0.27)***	0.34
All	1.00 (0.18)***	0.21	3.21 (0.22)***	0.66	2.20 (0.23)***	0.45
GAD-7 adj.^a^						
Males	-0.03 (0.24)	-0.01	1.42 (0.39)***	0.31	1.40 (0.41)***	0.32
Females	0.16 (0.23)	0.03	1.06 (0.26)***	0.21	0.77 (0.22)***	0.16
All	0.03 (0.17)	0.01	1.09 (0.22)***	0.21	0.91 (0.20)***	0.18
SMFQ crude						
Males	0.74 (0.28)**	0.15	3.01 (0.44)***	0.60	2.27 (0.51)***	0.43
Females	-0.12 (0.34)	-0.02	2.81 (0.38)***	0.43	2.93 (0.33)***	0.48
All	1.19 (0.22)***	0.21	4.45 (0.27)***	0.74	3.27 (0.28)***	0.54
SMFQ adj. ^a^						
Males	0.09 (0.25)	0.02	1.39 (0.40)***	0.28	1.30 (0.44)**	0.25
Females	-0.05 (0.30)	-0.01	2.15 (0.35)***	0.33	1.96 (0.29)***	0.31
All	-0.01 (0.19)	0.00	1.83 (0.26)***	0.29	1.74 (0.24)***	0.28
WEMWBS crude						
Males	-0.74 (0.57)	-0.08	-2.53 (0.89)**	-0.25	-1.78 (0.89)*	-0.20
Females	0.77 (0.53)	0.08	-2.13 (0.58)***	-0.21	-2.89 (0.51)***	-0.31
All	-1.32 (0.39)***	-0.13	-4.69 (0.45)***	-0.46	-3.38 (0.44)***	-0.36
WEMWBS adj. ^a^						
Males	-0.46 (0.48)	-0.05	-0.78 (0.77)	-0.08	-0.62 (0.74)	-0.07
Females	0.14 (0.45)	0.02	-1.81 (0.51)***	-0.18	-1.82 (0.42)***	-0.19
All	-0.19 (0.33)	-0.02	-1.45 (0.42)***	-0.14	-1.40 (0.36)***	-0.15

^*^p < .05, **p < .01, ***p < .001

^a^Models adjusted for socioeconomic status, frequency and duration of social media use, extraversion, and emotional stability. Models including both genders also adjusted for gender

The post-hoc analysis showed that the correlation coefficient was 0.38 (*p* < .001) for the SPAUSCIS and symptoms of depression, 0.36 (*p* < .001) for SPAUSCIS and symptoms of anxiety, and -0.27 (*p* < .001) for well-being.

### Longitudinal associations

First difference modelling was used to assess how changes in focus on self-presentation, measured by the SPAUSCIS, from T1 to T2 was related to changes in symptoms of anxiety and depression, and well-being. The first difference model yielded a coefficient of 0.85 (SD 0.36, *p* = .037) for symptoms of anxiety, 1.53 (SD 0.39, *p* < .001) for symptoms of depression, and a non-significant coefficient for well-being (-1.24, SD 0.68, *p* = .069). Thus, for each increase of 1 on the SPAUSCIS (total score ranging from 1 to 5) from T1 to T2, symptoms of anxiety increased by 0.85 and symptoms of depression increased by 1.53. The decrease in well-being for each increase of 1 on the SPAUSCIS scale did not reach statistical significance. Using standardized coefficients, each increase of 1 on the SPAUSCIS from T1 to T2 was associated with an increase of 0.17 standard deviations in symptoms of anxiety (SD 0.07, *p* < .05) and 0.25 standard deviations in symptoms of depression (SD 0.06, *p* < .001), both corresponding to small effect sizes [44], and a non-significant decrease of -0.13 (SD 0.07, *p* = .069) in well-being.

## Discussion

In this study, we used both cross-sectional and longitudinal data to investigate the relationship between focus on self-presentation on social media and experiences of symptoms of depression, anxiety, and overall well-being among adolescents. The results of a latent class analysis revealed, in line with a previous study [25], that the participants’ response patterns could be best characterized by a three-class solution, representing varying degrees of focus on self-presentation: low, intermediate, and high. A high focus on self-presentation was associated with higher scores on symptoms of depression and anxiety for both males and females, and lower scores on well-being among females, compared to a low or intermediate focus on self-presentation. Effect sizes ranged from small to medium. Additionally, we found that an increasing emphasis on self-presentation over time was associated with an increase in symptoms of anxiety and depression, although these effects were relatively small. Conversely, the association between an increased focus on self-presentation and well-being did not reach statistical significance. Hence, our findings suggest that a heightened focus on self-presentation, which includes behaviours like seeking feedback, employing strategic self-presentation tactics, and engaging in upward social comparisons, is associated with a small increase in risk of negative mental health outcomes.

Our findings are in line with previous studies [11, 18–21], and they add to the literature by showing these relationships by also using a longitudinal approach. Although this study is unable to establish a causal link between focus on self-presentation and mental health, there are some candidate mechanisms that could explain such a link. Firstly, placing importance on likes and comments may reflect a sense of self-worth that relies on online validation, making the individual vulnerable to fluctuations in likes and comments. Secondly, focus on self-presentation can be related to what Steele et al. [22] termed ‘approval anxiety’, which can contribute to an overall stress reaction (‘digital stress’) and consequently lead to symptoms of anxiety and depression, and lower mental well-being. Thirdly, a high focus on self-presentation may reflect a higher level of self-objectifications, i.e., an internalization of the observers’ gaze and viewing oneself as an object [45], which is regarded a risk factor for mental health problems [46–48]. Conversely, it is also possible that mental health problems lead to a higher focus on self-presentation. Studies have shown that underlying risk factors for poor mental health, such as shyness, loneliness, and neuroticism, predict heavier social media use and problematic social media use [49, 50], and may also predict a higher focus on self-presentation. To disentangle the causal relationship between focus on self-presentation and mental health, large multi-wave longitudinal studies are needed. The current finding that focus on self-presentation and mental health problems change concurrently indicate a crucial avenue for further investigation.

The current results showed that the group with a high focus on self-presentation was dominated by girls, but that the associations between focus on self-presentation and depression, anxiety, and well-being were not statistically different for males and females, as indicated by the interaction analysis. Similarly, Maheux et al. [24] found that while girls reported a higher level of preoccupation with their physical attractiveness in social media photos, the longitudinal association with depressive symptoms were similar for boys and girls. In the fully adjusted models of the present study, however, well-being was only associated with class membership for the sample as a whole and for girls, but not for boys. This may be related to the overrepresentation of girls in the dataset or to unobserved variables that are affecting the association differently for each gender. In the present study, we had no information about the content of the participants’ self-presentation. Studies have shown that girls’ self-presentation differs from boys. For example, girls have been shown to post more selfies and be more invested in physical appearance [26], which may impact the association between focus on self-presentation and well-being. A study by Svensson, Johnson, & Olsson [51] also showed gender differences, finding that self-presentation was associated with internalizing symptoms for girls only. Future research should explore these gender differences in the interactions between aspects of social media use and well-being.

In our study, no longitudinal association was observed between focus on self-presentation and well-being. This suggests that while an increase in focus on self-presentation over time may be related to more symptoms of anxiety and depression, it does not appear to impact well-being. The small effect sizes found for symptoms of anxiety and depression support this notion. At the same time, our longitudinal sample size was relatively limited, which may have contributed to the nonsignificant association due to issues of statistical power. Furthermore, it is possible that focus on self-presentation does not change very much from one year to the next, and that a longer time span would yield larger differences between focus on self-presentation at baseline and follow-up and a clearer relationship between focus on self-presentation and mental health and well-being. It is also possible that the relationship between focus on self-presentation and mental health differs between younger and older adolescent, in line with a study showing that the strength of the relationship between social media use and life satisfaction changed depending on the adolescents’ age [52]. Specifically, higher social media use predicted decreases in life satisfaction one year later among girls at ages 11–13 and 19, and among boys at 13–15 and 19. Exploring how focus on self-presentation is related to well-being among younger adolescents than those included in our study (16 +), would be of interest.

Our findings are in line with the results of a recent study by Winstone and colleagues [23], who employed latent class analysis to identify different user types on social media among 13-year-olds and how these types were related to mental health outcomes. In their study, adolescents characterized by high levels of content sharing (‘Broadcasters’) had a higher risk of poor mental health one year later, compared to those with moderate content sharing. In the present study, we did not measure self-presentation activity such as frequency of posting content, but rather how preoccupied the participants were with the feedback they received, strategic self-presentation, and their degree of upward social comparison. It may be that adolescents who post a lot on social media are also highly preoccupied with their online self-presentation, and that it is their preoccupation with self-presentation that increases their risk of mental health problems and not posting per se. In fact, some research indicates that self-presenting on social media even can have some benefits. For example, studies have shown that people can experience an increase in self-esteem after viewing one’s own social media profile [53, 54], and social media can facilitate authentic self-presentation of aspects of the self that are perceived as unwanted in offline social setting [55]. Furthermore, positive self-presentation (i.e., showing positive sides of the self) has been shown to increase subjective well-being, perhaps by supporting self-affirmation [56] and a positive self-image [57]. Future studies should explore these dynamics of posting on social media, different aspects of focus on self-presentation, and mental health in order to inform interventions to reduce mental health problems among adolescents.

### Implications

Only a high focus on self-presentation was associated with a higher risk of symptoms of depression and anxiety in fully adjusted analyses; an intermediate focus did not show this relationship. This finding aligns with other research showing that moderate use of social media is not linked to negative outcomes, and that only high use or high investment is [58–60]. For example, one study found that using visual social media such as Instagram and Snapchat for more than two hours each day positively predicted internalizing symptoms, while less than two hours of use each day did not [59]. This implies that the common notion that all social media use is negative for mental health is unwarranted and can lead to unnecessary worrying among adolescents about their social media use. However, the results of the present study suggest that a high focus on self-presentation may increase the risk of mental health problems, and helping adolescents balance their preoccupation with self-presentation, for example using school-based programs, should be a priority. School-based programs that encourages adolescents to reflect and think critically about their own and others’ use of social media, may facilitate higher levels of social media literacy and build their resilience and ability to leverage the potential positive effects of social media, while negating negative effects [61]. Furthermore, in a clinical context, dimensions of social media use such as feedback-seeking and strategic self-presentation are important topics to consider, as there is a possibility that they contribute to a worsening of mental health. However, as social media also give opportunities for social support and friendship formation, particularly for marginalized groups (e.g., [62]), an open-minded approach is warranted. Furthermore, tech producers could help minimize any negative effects of social media use by for instance limiting affordances that trigger upward social comparison and feedback-seeking, such as beauty filters and likes, thus redesigning their social media platforms to support, rather than harm, mental health.

### Strengths and limitations

In line with an affordances approach [4], this study focused on key attributes of social media platforms rather than specific social media platforms. Thus, the findings can be applicable to a wide range of platforms now and in the future. Furthermore, the measure of focus on self-presentation on social media was developed based on qualitative interviews with adolescents and adapted based on adolescent feedback, thus increasing the likelihood that the measure covered aspects of social media use that are relevant for adolescents and moving beyond quantity or frequency of self-presentation.

The present study also had some important limitations. Firstly, the validity of the observed longitudinal association rests on the assumption that there were no unobserved time-variant factors impacting the measured variables across the study period. The first difference model accounts for time-invariant factors such as gender, personality, and socioeconomic status, but factors that change over time are not accounted for. For example, given that the data were collected during the COVID-19 pandemic, it is possible that periods of lockdowns have led both to an increase in focus on self-presentation and in symptoms of depression and anxiety, and residual confounding cannot be ruled out. Secondly, the study included only two time points and is therefore limited in terms of determining cause and effect. Thirdly, assessing the relationship between focus on self-presentation and mental health and well-being over a longer time span could possibly have yielded a stronger relationship. To fully disentangle these causal relationships, further research employing large, multi-wave longitudinal studies is warranted. Furthermore, it is possible that the relationship between focus on self-presentation and mental health differs across different developmental periods, and studies should include a wider age range.

## Conclusion

This study employed both cross-sectional and longitudinal data to investigate the link between adolescents’ focus on self-presentation on social media and their symptoms of depression, anxiety, and overall well-being. Analysing the data using LCA, we identified three distinct groups characterized by varying degrees of self-presentation focus: low, intermediate, and high. A high focus on self-presentation was associated with more symptoms of anxiety and depression for boys and girls, and with lower well-being for girls in fully adjusted models. Furthermore, an increase in self-presentation focus over time was associated with small increases in depressive and anxiety symptoms, while the effect on well-being was not statistically significant. These findings suggest that a high focus on self-presentation, including behaviours like seeking feedback, strategic self-presentation, and upward social comparisons, is associated with an elevated risk of poor mental health. The observed covariance between that focus on self-presentation and mental health problems underscores a significant relationship warranting further investigation.

## Supplementary Information

Supplementary Material 1.

## Data Availability

The datasets analysed during the current study are not publicly available, as they contain sensitive information, and the ethical approval of the study does not include this option. Requests to access these datasets should be directed to GJH, Gunnhildjohnsen.Hjetland@fhi.no.

## Abbreviations

SPAUSCIS: Self-Presentation and Upwards Social Comparison Inclination Scale
GAD-7: General Anxiety Disorder 7
SMFQ: Short Mood and Feelings Questionnaire
WEMWBS: Warwick-Edinburgh Mental Well-Being Scale
SES: Socioeconomic status
LCA: Latent class analysis
CFA: Confirmatory factor analysis
