# Supplementary material for: Digital self-presentation and adolescent mental health: Cross-sectional and longitudinal insights from the “LifeOnSoMe”-study
Source: BMC Public Health. 2024 Sep 27;24:2635. doi: 10.1186/s12889-024-20052-4 (PMC11437887; doi:10.1186/s12889-024-20052-4)
Supplement: Supplementary file 1 — Supplementary Material 1. [file 12889_2024_20052_MOESM1_ESM.docx]

**Appendix**

Table S1: The Self-presentation and Upward Social Comparison Inclination Scale (SPAUSCIS)

1. I use a lot of time and energy on the content I post on social media

2. It is important to me that my posts receive many likes and/or comments

3. It is important to me to have many followers on social media

4. I delete posts on social media that do not receive enough likes and/or comments

5. I retouch images of myself to look better before I post them on social media

6. What others post on social media (images/status updates/stories) makes me feel less

content with myself and my life

7. The response I get for what I post (images/status updates/stories) impacts how I feel

Note: The response categories were “not at all”, “very little”, “sometimes/partly true”, “a

lot”, and “very much”, coded 1–5.


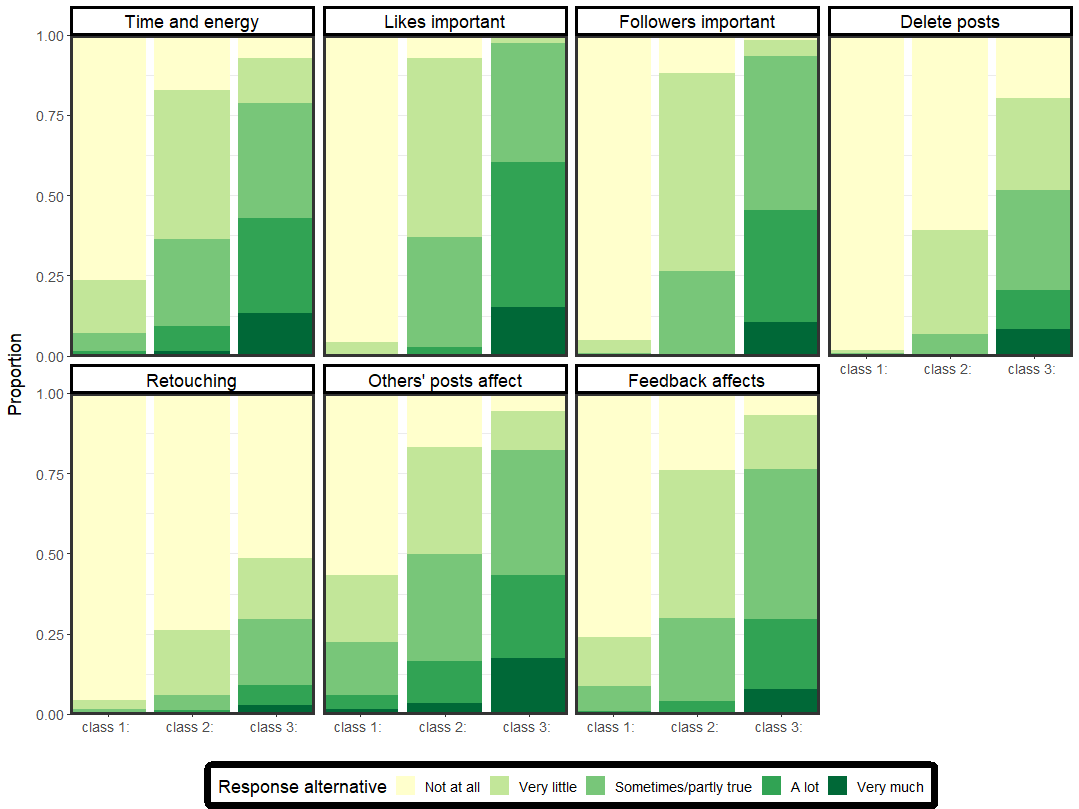


Figure S1 Response probabilities on the Self-presentation and upward social comparison inclination scale across retained classes

Table S2: LCA with 1-5 classes

|  | AIC | BIC | Relative entropy | LMR-LR |
| --- | --- | --- | --- | --- |
| 1 | 57709.19 | 57881.07 | - | - |
| 2 | 49501.20 | 49851.10 | 0.911 | p<.001 |
| *3* | *47331.16* | *47859.08* | *0.884* | *P<.001* |
| 4 | 46656.92 | 47362.85 | 0.878 | p=0.575 |
| 5 | 46214.41 | 47098.37 | 0.836 | p=0.760 |

Note. Data in italics indicates the best fitting model relative to the other models tested

AIC=Akaike information criterion; BIC=Bayesian information criterion; LMR-LR=Lo-Mendell-Rubin ad hoc adjusted likelihood ratio test.

Table S3: Descriptives across class membership

|  | Class 1 (N=1503) | Class 2 (N=1142) | Class 3 (N=779) | Total (N=3424) | p value |
| --- | --- | --- | --- | --- | --- |
| **Gender** |  |  |  |  | < 0.001^a^ |
| Male | 956 (63.6%) | 407 (35.6%) | 145 (18.6%) | 1508 (44.0%) |  |
| Female | 547 (36.4%) | 735 (64.4%) | 634 (81.4%) | 1916 (56.0%) |  |
| **Age** |  |  |  |  | 0.390 ^b^ |
| Mean (SD) | 17.30 (1.04) | 17.25 (0.94) | 17.27 (0.96) | 17.28 (0.99) |  |
| **Year of high school** |  |  |  |  | 0.070 ^a^ |
| 1 | 331 (22.1%) | 223 (19.6%) | 140 (18.0%) | 694 (20.4%) |  |
| 2 | 691 (46.2%) | 509 (44.8%) | 370 (47.6%) | 1570 (46.0%) |  |
| 3 | 474 (31.7%) | 405 (35.6%) | 267 (34.4%) | 1146 (33.6%) |  |
| **Study program** |  |  |  |  | < 0.001 ^a^ |
| College preparatory | 1030 (68.9%) | 908 (79.6%) | 616 (79.1%) | 2554 (74.8%) |  |
| Vocational education | 464 (31.1%) | 232 (20.4%) | 163 (20.9%) | 859 (25.2%) |  |
| **Country of birth** |  |  |  |  | 0.532 ^a^ |
| Norway | 1364 (90.9%) | 1046 (91.6%) | 701 (90.1%) | 3111 (90.9%) |  |
| Other country | 137 (9.1%) | 96 (8.4%) | 77 (9.9%) | 310 (9.1%) |  |
| **SES** |  |  |  |  | 0.004 ^a^ |
| Low (0-4) | 81 (5.5%) | 73 (6.4%) | 58 (7.5%) | 212 (6.3%) |  |
| Medium (5-7) | 729 (49.4%) | 607 (53.5%) | 427 (55.1%) | 1763 (52.1%) |  |
| High (8-10) | 666 (45.1%) | 454 (40.0%) | 290 (37.4%) | 1410 (41.7%) |  |
| **Frequency of social media use** |  |  |  |  | < 0.001 ^a^ |
| Daily or less | 520 (34.6%) | 216 (18.9%) | 88 (11.3%) | 824 (24.1%) |  |
| Many times each day | 705 (47.0%) | 622 (54.5%) | 373 (47.9%) | 1700 (49.7%) |  |
| Almost constantly | 276 (18.4%) | 304 (26.6%) | 318 (40.8%) | 898 (26.2%) |  |
| **Duration of social media use** |  |  |  |  | < 0.001 ^a^ |
| <2 hours | 554 (37.1%) | 311 (27.4%) | 148 (19.0%) | 1013 (29.7%) |  |
| 2-4 hours | 548 (36.7%) | 466 (41.0%) | 281 (36.1%) | 1295 (38.0%) |  |
| 4-5 hours | 208 (13.9%) | 218 (19.2%) | 188 (24.2%) | 614 (18.0%) |  |
| >5 hours | 184 (12.3%) | 142 (12.5%) | 161 (20.7%) | 487 (14.3%) |  |
| **Anxiety** |  |  |  |  | < 0.001 ^b^ |
| Mean (SD) | 472 (4.73) | 5.72 (4.62) | 7.92 (5.20) | 5.78 (4.96) |  |
| **Depression** |  |  |  |  | < 0.001 ^b^ |
| Mean (SD) | 5.83 (5.69) | 7.02 (5.62) | 10.28 (6.61) | 7.24 (6.14) |  |
| **Well-being** |  |  |  |  | < 0.001 ^b^ |
| Mean (SD) | 49.96 (10.36) | 48.65 (9.10) | 45.27 (9.97) | 48.46 (10.03) |  |

^a^Pearson's Chi-squared test

^b^ Linear Model ANOVA

Table S4: Response rates on the items of the Self-presentation and upward social comparison inclination scale (SPAUSCIS) across class membership

|  | Class 1 (N=1503) | Class 2 (N=1142) | Class 3 (N=779) | Total (N=3424) | p value |
| --- | --- | --- | --- | --- | --- |
| **1. Time and energy** |  |  |  |  | < 0.001^a^ |
| N-Miss | 9 | 9 | 10 | 28 |  |
| Not at all | 1142 (76.4%) | 197 (17.4%) | 52 (6.8%) | 1391 (41.0%) |  |
| Very little | 245 (16.4%) | 532 (47.0%) | 99 (12.9%) | 876 (25.8%) |  |
| Sometimes/partly true | 86 (5.8%) | 304 (26.8%) | 283 (36.8%) | 673 (19.8%) |  |
| A lot | 16 (1.1%) | 85 (7.5%) | 229 (29.8%) | 330 (9.7%) |  |
| Very much | 5 (0.3%) | 15 (1.3%) | 106 (13.8%) | 126 (3.7%) |  |
| **2. Likes important** |  |  |  |  | < 0.001^a^ |
| N-Miss | 10 | 11 | 9 | 30 |  |
| Not at all | 1442 (96.6%) | 65 (5.7%) | 4 (0.5%) | 1511 (44.5%) |  |
| Very little | 47 (3.1%) | 641 (56.7%) | 13 (1.7%) | 701 (20.7%) |  |
| Sometimes/partly true | 4 (0.3%) | 398 (35.2%) | 280 (36.4%) | 682 (20.1%) |  |
| A lot | 0 (0.0%) | 27 (2.4%) | 355 (46.1%) | 382 (11.3%) |  |
| Very much | 0 (0.0%) | 0 (0.0%) | 118 (15.3%) | 118 (3.5%) |  |
| **3. Followers important** |  |  |  |  | < 0.001^a^ |
| N-Miss | 11 | 8 | 9 | 28 |  |
| Not at all | 1427 (95.6%) | 129 (11.4%) | 11 (1.4%) | 1567 (46.1%) |  |
| Very little | 51 (3.4%) | 705 (62.2%) | 34 (4.4%) | 790 (23.3%) |  |
| Sometimes/partly true | 14 (0.9%) | 300 (26.5%) | 372 (48.3%) | 686 (20.2%) |  |
| A lot | 0 (0.0%) | 0 (0.0%) | 271 (35.2%) | 271 (8.0%) |  |
| Very much | 0 (0.0%) | 0 (0.0%) | 82 (10.6%) | 82 (2.4%) |  |
| **4. Delete posts** |  |  |  |  | < 0.001^a^ |
| N-Miss | 17 | 9 | 11 | 37 |  |
| Not at all | 1456 (98.0%) | 700 (61.8%) | 137 (17.8%) | 2293 (67.7%) |  |
| Very little | 17 (1.1%) | 363 (32.0%) | 224 (29.2%) | 604 (17.8%) |  |
| Sometimes/partly true | 9 (0.6%) | 65 (5.7%) | 247 (32.2%) | 321 (9.5%) |  |
| A lot | 3 (0.2%) | 4 (0.4%) | 96 (12.5%) | 103 (3.0%) |  |
| Very much | 1 (0.1%) | 1 (0.1%) | 64 (8.3%) | 66 (1.9%) |  |
| **5. Retouching** |  |  |  |  | < 0.001^a^ |
| N-Miss | 18 | 11 | 7 | 36 |  |
| Not at all | 1418 (95.5%) | 844 (74.6%) | 388 (50.3%) | 2650 (78.2%) |  |
| Very little | 45 (3.0%) | 225 (19.9%) | 149 (19.3%) | 419 (12.4%) |  |
| Sometimes/partly true | 18 (1.2%) | 48 (4.2%) | 164 (21.2%) | 230 (6.8%) |  |
| A lot | 2 (0.1%) | 11 (1.0%) | 49 (6.3%) | 62 (1.8%) |  |
| Very much | 2 (0.1%) | 3 (0.3%) | 22 (2.8%) | 27 (0.8%) |  |
| **6. Others’ posts affect** |  |  |  |  | < 0.001^a^ |
| N-Miss | 49 | 42 | 33 | 124 |  |
| Not at all | 818 (56.3%) | 193 (17.5%) | 38 (5.1%) | 1049 (31.8%) |  |
| Very little | 310 (21.3%) | 359 (32.6%) | 90 (12.1%) | 759 (23.0%) |  |
| Sometimes/partly true | 243 (16.7%) | 368 (33.5%) | 287 (38.5%) | 898 (27.2%) |  |
| A lot | 60 (4.1%) | 145 (13.2%) | 196 (26.3%) | 401 (12.2%) |  |
| Very much | 23 (1.6%) | 35 (3.2%) | 135 (18.1%) | 193 (5.8%) |  |
| **7. Feedback affects** |  |  |  |  | < 0.001^a^ |
| N-Miss | 54 | 43 | 36 | 133 |  |
| Not at all | 1094 (75.5%) | 271 (24.7%) | 48 (6.5%) | 1413 (42.9%) |  |
| Very little | 228 (15.7%) | 502 (45.7%) | 121 (16.3%) | 851 (25.9%) |  |
| Sometimes/partly true | 112 (7.7%) | 280 (25.5%) | 352 (47.4%) | 744 (22.6%) |  |
| A lot | 12 (0.8%) | 39 (3.5%) | 163 (21.9%) | 214 (6.5%) |  |
| Very much | 3 (0.2%) | 7 (0.6%) | 59 (7.9%) | 69 (2.1%) |  |
| **SPAUSCIS total** |  |  |  |  | < 0.001^b^ |
| Mean (SD) | 1.22 (0.25) | 2.02 (0.33) | 3.09 (0.51) | 1.91 (0.81) |  |

^a^Pearson's Chi-squared test

^b^ Linear Model ANOVA

Table S5 Likelihood ratio tests comparing models with (model 1) and without (model 0) the interaction term class membership*gender

|  | Model 1 vs model 0,  p-value |
| --- | --- |
| Symptoms of anxiety | .323 |
| Symptoms of depression | .171 |
| Well-being | .151 |
